# The relationship between yttrium-90 glass microspheres specific activity, particle density and treatment outcomes in HCC and mCRC

**DOI:** 10.1007/s00259-025-07334-8

**Published:** 2025-05-22

**Authors:** Marnix G. E. H. Lam, Etienne Garin, Kirk D. Fowers, Armeen Mahvash, Siddharth A. Padia, Riad Salem

**Affiliations:** 1 Department of Radiology and Nuclear Medicine, University Medical Center Utrecht, Huispostnummer E01.132, Postbus 85500, 3508 GA Utrecht, The Netherlands; 2 Nuclear Medicine Department, Eugene Marquis Center, Univ Rennes, INSERM, INRA, Centre de Lutte Contre Le Cancer Eugène Marquis, Institut NUMECAN (Nutrition Metabolisms and Cancer), 35000 Rennes, France; 3 Boston Scientific Corporation, Marlborough, MA USA; 4 Department of Interventional Radiology, University of Texas MD Anderson Cancer Center, Houston, TX USA; 5 Department of Radiology, University of California-Los Angeles, Los Angeles, CA USA; 6 Department of Radiology, Northwestern University Feinberg School of Medicine, Chicago, IL USA

**Keywords:** Radioembolization, Yttrium-90, Dose, Specific activity, Particle density

## Abstract

**Purpose:**

To investigate relationships between treatment week, relative to Ytrrium-90 (^90^Y) glass microsphere calibration (i.e., specific activity and particle density), and outcomes for hepatocellular carcinoma (HCC) or colorectal cancer liver metastasis (mCRC).

**Methods:**

Multinational, multicenter study TARGET (retrospective; *n* = 209 HCC patients) was combined with EPOCH (phase III trial; *n* = 428 mCRC patients). Efficacy included overall response rate (ORR), overall survival (OS), progression-free survival (PFS), hepatic PFS, and tumour marker response rates. Safety included clinical and laboratory toxicity. Retrospective multicompartment dosimetry, tumour and normal tissue absorbed dose were available for TARGET; single compartment dosimetry was available for EPOCH.

**Results:**

No efficacy relationship was found relative to treatment week for TARGET or EPOCH. mRECIST ORR in TARGET for weeks 1 and 2 were 74/125 (59.2%) and 55/84 (65.5%), and by RECIST 1.1 in EPOCH were 54/142 (38.0%) and 15/43 (34.9%), respectively (*p* > 0.05). Median OS for TARGET weeks 1 and 2 were 21.4 and 20.3 months (*p* = 0.07), and in EPOCH were 14.9 and 16.4 months, respectively (*p* = 0.37). No difference in the TARGET primary endpoint of hyperbilirubinemia was noted for weeks 1 and 2, odds ratio 0.64, *p* = 0.59. TARGET ≥ grade 3 device-related adverse events (AEs) for weeks 1 (16.8%) and 2 (26.2%) were not significantly different (*p* = 0.11). EPOCH rates of ≥ grade 3 asthenia for weeks 1 (9.2%) and 2 (23.3%) were statistically different (*p* = 0.01).

**Conclusions:**

No efficacy treatment benefit for week 2 versus week 1 was observed in TARGET or EPOCH, but week 2 treatment trended towards a higher rate and severity of specific AEs.

**Supplementary Information:**

The online version contains supplementary material available at 10.1007/s00259-025-07334-8.

## Introduction

Transarterial radioembolization (TARE) is a well-established loco-regional treatment option for primary and secondary liver cancer. Intra-arterial distribution of Yttrium-90 (^90^Y) microspheres aims to maximize the tumour absorbed dose (TAD) while minimizing the normal tissue absorbed dose (NTAD). Compared with other currently available TARE products, ^90^Y glass microspheres (TheraSphere™, Boston Scientific Corporation, Marlborough, MA, USA) have a high specific activity (amount of radioactivity per microsphere) at calibration. At calibration, ^90^Y glass microspheres have a specific activity of 4,000 ± 400 Bq/microsphere, whereas on second week Monday (eight days after calibration), the same microspheres have a specific activity of approximately 500 Bq/microsphere (Boston Scientific data on file) [1, 2]. Dose depends on the specific activity and the number of microspheres at the time of treatment, which are inversely related. Upon optimization of two variables (i.e., dose and specific activity), the third will be fixed (i.e. number of microspheres). Thus, to reach a specific TAD, e.g., 300 Gy, increasingly more microspheres are required as the time from calibration increases, due to decreasing specific activity. The absorbed dose distribution depends on 1) injected activity, 2) treatment perfused volume, and 3) the relative distribution of microspheres in tumour and normal tissue. While technique plays a role, the number of microspheres in the perfused volume (particle density) is the dominant factor determining distribution. For a fixed mean absorbed dose, the specific activity determines the microsphere number/mL (particle density) [1]. Absorbed dose distribution may be quantified by dose volume histogram (DVH), e.g., D_70_ reflects the absorbed dose that 70% of the tumour receives [3, 4]. Particle density pertains to the spatial microsphere distribution, where a minimum number of ^90^Y glass microspheres are required to achieve sufficient tumour absorbed dose distribution by DVH [5]. The difference in specific activity of the administered microspheres, ranging from high specific activity in the first week after calibration to lower specific activity at the end of week 2 for ^90^Y glass microspheres, is an important factor that determines microsphere distribution and therefore dose thresholds relative to safety and efficacy [6].

Heterogeneity of tumour distribution is cited as one rationale for requiring a higher TAD to achieve therapeutic efficacy, due to heterogenous tumour vascularization [4, 5]. In a study of larger tumours (≥ 5 cm), tumours achieving complete response (CR) had higher mean TADs than tumours without CR, and higher V100 was significantly associated with CR by multivariate analysis [7]. Multiple studies have demonstrated that below a threshold, distribution affects coverage. However, above a threshold of ~ 10,000 microspheres/mL, injecting more microspheres does not change heterogenous microsphere distribution within the tumour [1, 6, 8]. Indeed, preferential tumour microsphere distribution was demonstrated using magnetic resonance imaging after injecting multiple dose fractions, which did not show significant changes in heterogeneous microsphere distribution with subsequent doses [8]. At the same time, heterogeneity is also cited as the reason behind reduced normal tissue toxicity due to less homogenous radiation delivery [4, 9, 10]. Differences in the heterogenous distribution of ^90^Y glass and resin microspheres (SIR-Spheres, Sirtex) is also noted as the reason for ^90^Y glass having a higher tolerable NTAD, approximately 70–80 Gy versus 40 Gy [9, 11].

Institutional practice differs in selection of treatment day for ^90^Y glass microspheres as reflected in the current hepatocellular carcinoma (HCC) expert panel recommendations, which noted clinical data supporting treatment from week 1 Wednesday through week 2 Tuesday [12]. The rationale for using more microspheres is based on a hypothesis that it results in more homogenous distribution and achieves improved patient outcomes, however, supporting clinical data using a higher or lower number of microspheres at a comparable TAD is lacking. Rather, within the treatment recommendation window of week 1 Wednesday through week 2 Tuesday, increasing TAD above the minimum microsphere number increases the likelihood of CR and/or complete pathologic necrosis (CPN). The difference in target TAD and maximum NTAD for the two ^90^Y microsphere products highlights the potential impact of treatment week selection for ^90^Y glass microspheres. In addition to limited data showing improved efficacy with a higher number of microspheres, the potential toxicity to normal tissue has been relatively neglected. The present analyses evaluated whether the main argument that TAD is the primary driver for patient efficacy outcomes was confirmed when comparing treatment week in two large clinical studies, TARGET for HCC and EPOCH for colorectal carcinoma liver metastasis (mCRC).

## Materials & methods

### Theoretical model

Microsphere deposition and dose distributions in normal tissue and tumour were simulated. The total perfused volume (1500 mL) was modelled as a cylinder, with 1150 mL of normal tissue and a 350 mL spherical tumour. This corresponds with an 8.7 cm tumour diameter, and a perfused volume cylinder with a diameter and length of 12.4 cm. For V30 calculations we assumed the whole liver volume was 2250 mL (1900 mL of normal tissue and 350 mL of tumour).

The partition method with a target TAD and prespecified tumour to normal ratio (T:N) [13] was used to determine the delivered activity. The number of microspheres simulated in the model was computed from the delivered activity and microsphere specific activity. Microsphere deposition location in the tissue compartments was simulated with a stochastic model based on normal distribution following a prespecified T:N. Dose distributions resulting from the microsphere deposition locations were computed using an analytic dose distribution kernel. Probability of microsphere deposition at each location (voxel) in tumour and normal tissue was based on a randomly assigned probability of microsphere deposition following a normal distribution and a clinically observed heterogeneity index (HI), defined as D5/D95 [5]. Ten simulations were run for each treatment and the results were averaged.

### Clinical data

Patient datasets comprised of the study populations from TARGET and EPOCH who received ^90^Y glass microsphere treatment [14, 15]. TARGET was an international, multicenter, retrospective investigation in HCC patients from 13 centers across eight countries. EPOCH was an international, multicenter, open label randomized controlled phase III trial in patients with mCRC who progressed on first-line systemic therapy. TARGET and EPOCH represent the largest HCC and mCRC populations using ^90^Y glass microspheres published to date and offer an opportunity to evaluate the impact of treatment week on safety and efficacy outcomes. Dosimetry models used in TARGET and EPOCH allowed for the assessment of the impact of treatment week, and resultant inherent difference in specific activity of microspheres, on patient outcomes. See Table 1 for more details on specific activities used in TARGET and EPOCH studies. Both studies included patients requiring either lobar or bilobar treatment to allow assessment of toxicity and efficacy outcomes.

**Table 1:** Treatment week specific activities for TARGET and EPOCH studies

	Week 1 (Mon–Sun)	Week 2 (Mon–Fri)
TARGET		
Treatment day	4 (2–7)	8 (8–12)
Specific activity (Bq/microsphere)	1,417 (650–2,380)	502 (178–502)
Total activity (GBq)	2.83 (0.8–9.3)	2.55 (0.8–5.5)
EPOCH		
Treatment day	4 (1–7)	9 (8–12)
Specific activity (Bq/microsphere)	1,417 (650–3,086)	387 (178–502)
Total activity (GBq)	3.90 (0.6–11.7)	3.08 (0.4–15.0)

Data represented as median (range)

### Dosimetry

For single and multicompartment dosimetry models in TARGET, [^99 m^Tc]Tc-MAA was used as a surrogate to approximate the post-treatment distribution of ^90^Y glass microspheres. The target threshold absorbed dose using single compartmental dosimetry was per the ^90^Y glass microspheres Instructions for Use. Multicompartmental dosimetry thresholds for TAD and NTAD were guided by recent publications [15–17]. In EPOCH, absorbed dose of the perfused liver volume was assessed using single compartment dosimetry with the per protocol perfused volume absorbed dose specified as 120 Gy ± 10% [14]. Single compartment dosimetry assumes equal distribution of microspheres within the entire volume of perfused liver with microspheres (perfused volume) thus only perfused liver volume is required when calculating dose requirements.

### Treatment day

To evaluate the impact of microsphere number on treatment outcomes, treatment day was selected as the proxy for microsphere number. Glass microspheres are always calibrated on Sundays at noon, Eastern Time, and have a 12-day shelf life. Treatment day was assigned as week 1 (day of calibration zero to seven) or week 2 (days from calibration eight to 12). TARGET patients were further subdivided into early week 1 (Mon – Wed), late week 1 (Thu – Sun), early week 2 (Mon – Tue) and late week 2 (Wed – Fri). TARGET patients received either week 1 or week 2 ^90^Y glass microspheres. EPOCH patients who received more than one vial were subdivided into week 1 only, week 2 only or a combination of week 1 and week 2. If a patient received both a week 1 and week 2 vial, then week allocation was based on which week the largest perfused volume was received. This was typically based on right lobe treatment as most patients randomized to the ^90^Y plus systemic chemotherapy arm (80.5%) in EPOCH had bilobar disease for which whole liver treatment in a single session was the protocol specified treatment. Treatment day was at the discretion of the physician for both TARGET and EPOCH. In TARGET the choice of single or multicompartment dosimetry and the absorbed dose target(s) were at the discretion of the physician, however, retrospective analyses of the imaging data used multicompartmental dosimetry.

### Endpoints

Post-hoc analysis evaluated the effect of treatment day on safety and efficacy outcomes. For TARGET, the outcomes assessed were: adverse events (AEs) up to 90 days, by the National Cancer Institute’s Common Terminology (CTCAE) version 4.02; overall survival (OS); objective response rate (ORR) by mRECIST; and alpha-fetoprotein (AFP) response (≥ 50% reduction in patients with AFP > 200 ng/mL at baseline). For EPOCH, the outcomes assessed were: AEs every eight weeks from randomization, by CTCAE version 3.0; OS; progression-free survival (PFS); hepatic PFS (hPFS); ORR by RECIST 1.1; and carcinoembryonic antigen (CEA) response.

## Results

### Theoretical model

**Fig. 1:**
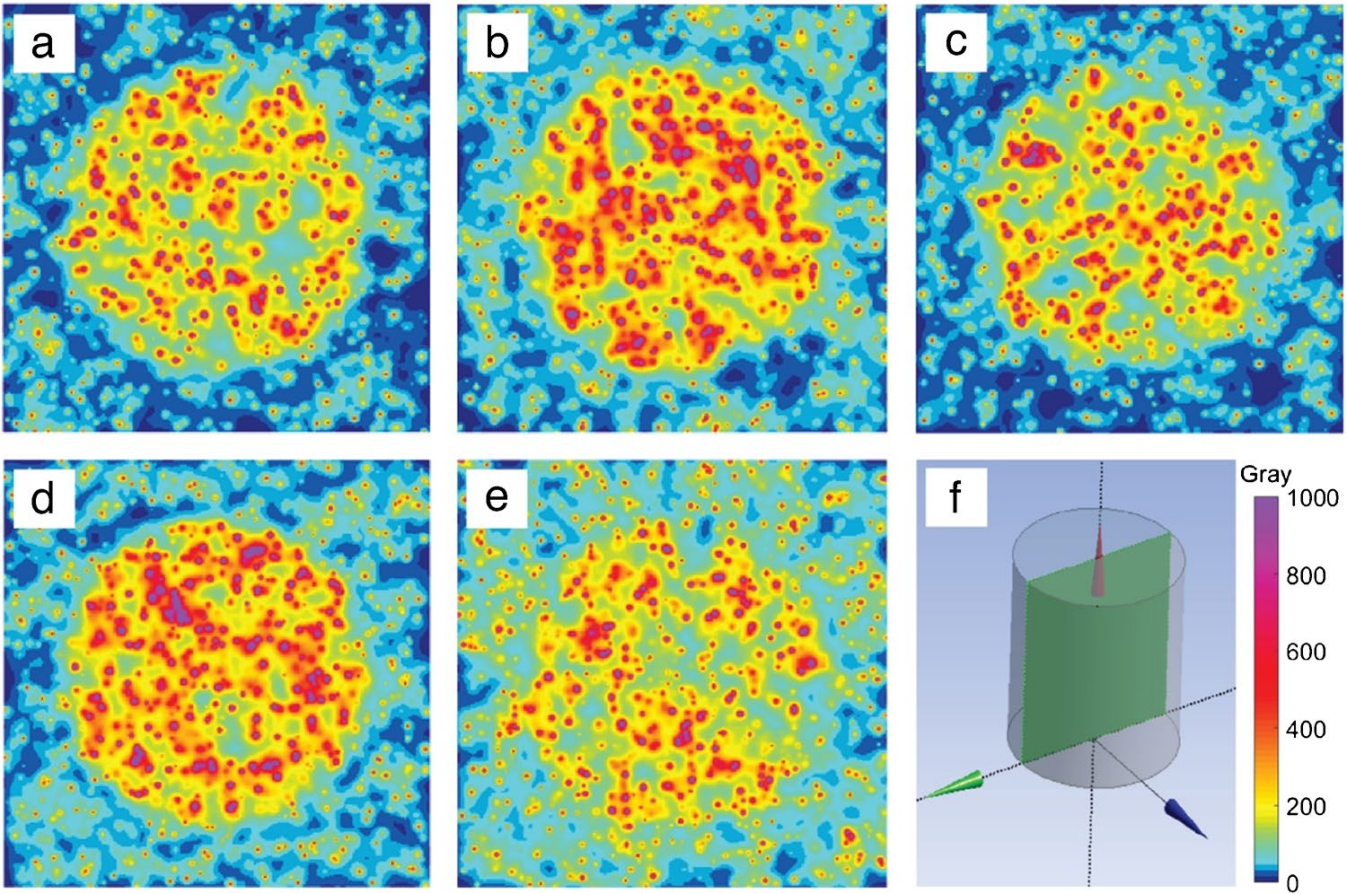
Microsphere distribution modelling of ^90^Y glass microspheres (4,000 Bq/microsphere at calibration). **a**) First week Wednesday targeting TAD at 250 Gy (1,836 Bq/microsphere); **b**) First week Wednesday targeting 350 Gy (1,836 Bq/microsphere); **c**) Second week Tuesday targeting 250 Gy (387 Bq/microsphere); **d**) Second week Tuesday targeting 350 Gy (387 Bq/microsphere); **e**) Second week Friday targeting 250 Gy (178 Bq/microsphere). **f**) Diagram of cross-section image and scale (Gy) used for a – e. The modelled T:N ratio was 4.9 for a, b, c and d and 2.4 for e

Figure 1 illustrates modelling of ^90^Y glass microsphere distribution as a function of a threshold TAD and treatment day, ranging from high specific activity in the first week after calibration to low specific activity at the end of week 2, at two TAD thresholds.

A higher specific activity requires a lower number of microspheres to achieve a specific TAD, resulting in differences in distribution (Fig. 1a versus Fig. 1c, and Fig. 1b versus Fig. 1d). For the same absorbed dose, a higher specific activity results in a higher distribution heterogeneity in both tumour and normal tissue in the model. For example, in normal tissue, higher specific activity results in a more heterogeneous normal tissue exposure, i.e., more hepatocytes receive higher radiation dose on one hand, but more hepatocytes are spared on the other hand. At treatment day 12, the administered ^90^Y glass microsphere distribution may theoretically be more homogeneous but may also lead to a decrease in T:N ratio (may be due to changes in tumour uptake into normal tissue i.e., overflow) resulting in increased NTAD (Fig. 1e).

**Fig. 2:**
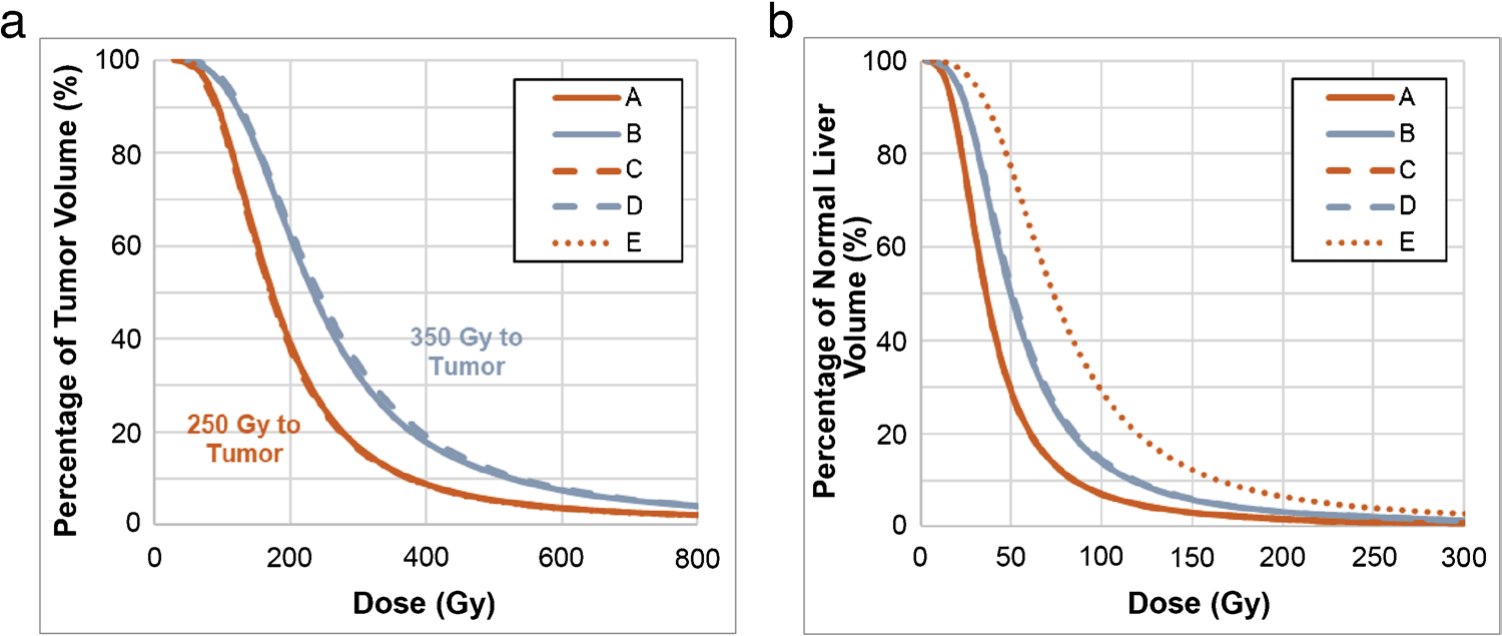
Tumour absorbed dose volume histogram (DVH) modelling of ^90^Y glass microspheres (4,000 Bq/microsphere at calibration): **a**) Tumour DVH for examples in Fig. 1a–e, b): Normal tissue DVH for examples in Fig. 1a–e

DVH modelling notes minimal changes in the shape of the DVH curve for week of administration (Fig. 2). Increasing the TAD threshold demonstrates a substantial TAD increase across the DVH curves (shift to the right), while a (dynamic) modelled decrease in the T:N ratio exhibits the largest increases in NTAD.

Table 2 provides example specific values for activity administered, number of microspheres administered, TAD (mean), NTAD (mean), TAD DVH D_95_ and D_5_, tumour and normal tissue HI, tumour V_0–100_ Gy, Normal V30 and tumour and normal tissue microspheres/mL.

**Table 2:** Theoretical model details for examples A to E

Example	Activity administered (GBq)	Number of microspheres (millions)	Specific Activity (Bq)^a^	TAD (Gy), mean (SD^b^)	NTAD (Gy), mean (SD^b^)	TAD D_95_ (Gy), (SD^b^)	TAD D_5_ (Gy), (SD^b^)	Tumour HI, (SD^b^)	Normal HI, (SD^b^)	Tumour V_0–100,_ % (SD^b^)	Normal V30 % (SD^b^)	Tumour Microspheres/mL	Normal Tissue Microspheres/mL
A	2.9	1.6	1836	230 (9)	50 (2)	78 (4)	517 (25)	6.6 (0.4)	8.1 (0.6)	13 (2)	38 (2)	2,720	555
B	4.1	2.2	1836	316 (14)	68 (3)	100 (7)	724 (45)	7.3 (0.7)	8.0 (0.4)	5 (2)	50 (2)	3,810	778
C	2.9	7.5	387	227 (10)	49 (1)	75 (4)	512 (34)	6.8 (0.6)	8.1 (0.8)	14 (2)	39 (1)	12,905	2,634
D	4.1	10.6	387	323 (15)	69 (1)	105 (10)	735 (42)	7.0 (0.6)	8.0 (0.4)	4 (2)	51 (1)	18,075	3,689
E	4.2	23.3	178	228 (14)	98 (4)	79 (7)	509 (36)	6.4 (0.5)	7.6 (0.5)	13 (3)	57 (1)	28,118	11,716

The target TAD for examples a, c and e was 250 Gy, while for examples b and d it was 350 Gy, however, edge effects of the model result in a lower calculated TAD mean within the model

^a^Specific activity was 4,000 Bq/microsphere at time of calibration

^b^SD is calculated from 10 repeated simulations

D5, absorbed dose reached in 5% of the target tumour; D95, absorbed dose reached in 95% of the target tumour; HI, heterogeneity index (D5/D95); SD, standard deviation; V_0–100_, percent of tumour volume with an absorbed dose between 0 and 100 Gy; V30, Volume percent of normal liver receiving at least 30 Gy

#### TARGET study data

TARGET patient and disease characteristics for week 1 and week 2 are presented in Table 3. Patient and baseline disease characteristics for Early week 1, Late week 1, Early week 2 and Late week 2 are presented in Supplementary Table 1. Treatment for most patients in TARGET (184/209 [88.0%] patients) was within the HCC recommendation time frame [12].

**Table 3:** TARGET patient baseline disease characteristics (week 1 and week 2)

	week 1 (*N* = 125) *n* (%)	week 2 (*N* = 84) *n* (%)	*p*-value^a^
ECOG status			0.769^b^
0	82 (65.6)	53 (63.1)	
1	40 (32.0)	27 (32.1)	
2/3	3 (2.4)	4 (4.8)	
BCLC stage			0.734
A	18 (14.4)	9 (10.7)	
B	39 (31.2)	29 (34.5)	
C	68 (54.4)	46 (54.8)	
Child–Pugh status			0.170
A (5–6)	115 (92.0)	72 (85.7)	
B7	10 (8.0)	12 (14.3)	
Unilobar	85 (68.0)	63 (75.0)	0.352
Bilobar	40 (32.0)	21 (25.0)	
Target Lesion Longest Diameter (RECIST 1.1)			0.048
Median	8.00	6.90	
Mean (SD)	8.40 (3.59)	7.31 (2.68)	
≥ 3 cm to < 5 cm	24 (19.2)	17 (20.2)	0.07
≥ 5 to < 8 cm	36 (28.8)	36 (42.9)	
≥ 8 cm	65 (52.0)	31 (36.9)	
Target Lesion Longest Diameter (mRECIST)			0.9111
Median	7.00	6.90	
Mean (SD)	7.30 (3.21)	7.25 (2.70)	
< 3 cm	2 (1.6)	0	0.07
≥ 3 cm to < 5 cm	31 (24.8)	18 (21.4)	
≥ 5 to < 8 cm	38 (30.4)	37 (44.0)	
≥ 8 cm	48 (38.8)	29 (34.5)	
Missing	6 (4.8)	0	
Presence of PVT	39 (31.2)	30 (35.7)	0.549
Bilirubin			0.450
< 1.0 mg/dL	82 (65.6)	60 (71.4)	
≥ 1.0 mg/dL	43 (34.4)	24 (28.6)	
Albumin			0.110
< 3.6 g/dL	41 (32.8)	37 (44.0)	
≥ 3.6 g/dL	84 (67.2)	47 (56.0)	
Ascites			0.171
Absent	115 (92.0)	72 (85.7)	
Slight	10 (8.0)	12 (14.3)	
HCC Etiology			
Alcohol	35 (28.0)	13 (15.5)	0.044
Hepatitis C	31 (24.8)	38 (45.2)	0.003
Unknown	23 (18.4)	9 (10.7)	0.170
Hepatitis B	14 (11.2)	17 (20.2)	0.078
Non-alcoholic steatohepatitis	13 (10.4)	7 (8.3)	0.811
Other	8 (6.4)	5 (6.0)	1.000

^a^Fisher’s exact test

^b^Statistical analysis compared ECOG 0 and ECOG ≥ 1

SD, standard deviation

The perfused volume absorbed dose was ≤ 150 Gy in 100 (80.0%) and 65 (77.4%) patients for week 1 and week 2, respectively. Total perfused TAD and NTAD were somewhat higher, but not statistically different for week 2 (Table 4).

**Table 4:** TARGET TAD and NTAD for week 1 and week 2

	Week 1 (*N* = 125)	Week 2 (*N* = 84^b^)	*p*-value^a^
Median TAD (Range), Gy	209.4 (14.0, 1050.9)	230.8 (26.1, 1130.4)	0.266
Mean TAD (SD), Gy	246.9 (165.4)	265.7 (168.2)	
TAD (%) < 200 Gy/≥ 200 to < 300 Gy/≥ 300 Gy	44.0/28.8/27.2	42.9/25.0/32.1	
Geometric Mean TAD (95% CI), Gy	202.0 (180.6, 226.1)	223.6 (195.0, 256.4)	
Median NTAD (Range), Gy	86.2 (16.8, 270.1)	88.9 (25.1, 194.3)	0.939
Mean NTAD (SD), Gy	93.4 (46.63)	91.1 (36.79)	
Geometric Mean NTAD (95% CI), Gy	83.3 (76.85, 90.33)	83.7 (75.8, 92.5)	

^a^Comparison of geometric means by 2-sample t-test

^b^NTAD available for N = 83 patients

SD, standard deviation

**Fig. 3:**
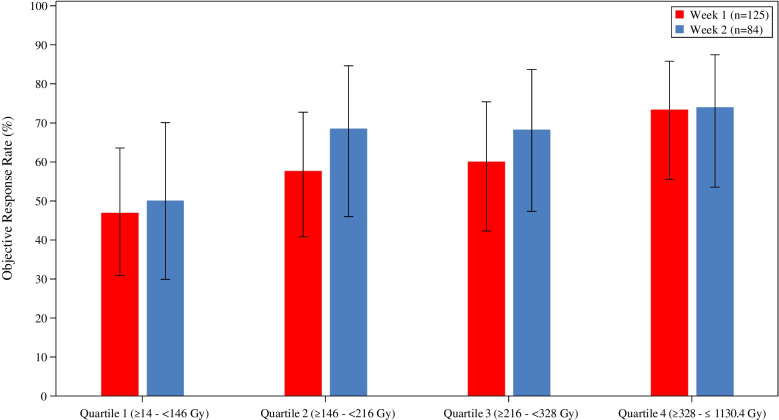
TARGET total perfused TAD by Quartile and ORR (mRECIST) over all patients (*n* = 209)

Objective response by mRECIST was achieved in 74 (59.2%) and 55 (65.5%) patients for week 1 and week 2, respectively. Logistic regression demonstrated no difference in ORR by mRECIST between week 1 and week 2 treatment, odds ratio = 1.27 (95% CI: 0.71–2.26), *p* = 0.43. However, increasing total perfused TAD demonstrated increasing ORR, regardless of week 1 versus week 2 treatment (Fig. 3). This was consistent with a multivariable logistic regression analysis, which showed greater ORR with higher total perfused TAD (odds ratio = 1.26 (95% CI: 1.02–1.55), *p* = 0.03).

Median OS was similar for week 1 (21.4 months, 95% CI: 15.6, 26.7) and week 2 (20.3 months, 95% CI: 16.6, 28.6), *p* = 0.73 by log rank test, and the week 2: week 1 hazard ratio (HR) was 1.07 (95% CI: 0.72, 1.60), *p* = 0.73, by Cox regression (Supplementary Fig. 1). Median OS was also similar for week 1 (Mon-Wed), week 1 (Thu-Sun), week 2 (Mon-Tue) and week 2 (Wed-Fri) (Supplementary Table 2 and Supplementary Fig. 2).

The AFP response, defined as a ≥ 50% reduction in AFP in patients with AFP > 200 ng/mL at baseline, was comparable for week 1 29/40 (72.5%) and week 2 (18/31) 58.1%, *p* = 0.22 by Fisher’s exact test.

No difference in the occurrence of ≥ grade 3 hyperbilirubinemia, the primary endpoint of TARGET, by logistic regression was noted between week 1 and week 2, odds ratio = 0.67 (95% CI: 0.17–2.70), *p* = 0.58.

Device related ≥ grade 3 events occurred in 21/125 week 1 patients (16.8%) and in 22/84 week 2 patients (26.2%) (*p* = 0.12). Specific ≥ grade 3 AEs by week 1 and week 2 are listed in Table 5. Further breakdown into treatment day windows within week 1 and week 2 are listed in Supplementary Table 3.

**Table 5:** TARGET device related ≥ grade 3 AEs by week of treatment

Adverse Events (n, %)^a^	week 1 (*N* = 125)	week 2 (*N* = 84)	*p*-value^b^
Patients with device related AEs ≥ grade 3	21 (16.8)	22 (26.2)	0.117
Abdominal pain	0	2 (2.4)	0.160
Ascites	3 (2.4)	7 (8.3)	0.093
Blood bilirubin increased	8 (6.4)	1 (1.2)	0.088
Hepatic enzyme increased	0	2 (2.4)	0.160
Lymphopenia	9 (7.2)	10 (11.9)	0.327
Thrombocytopenia	1 (0.8)	3 (3.6)	0.305
Oedema	1 (0.8)	2 (2.4)	0.566

^a^Adverse events in 2 or more patients (either week 1 or week 2) were included

^b^By Fisher’s exact test

### EPOCH study data

EPOCH data for 185 patients who were randomized to the ^90^Y glass microspheres plus systemic chemotherapy arm and who received ^90^Y prior to progression is presented here. EPOCH patient disease characteristics for week 1 and week 2 are presented in Table 6.

**Table 6:** EPOCH patient disease and treatment characteristics

	week 1 (*N* = 142) n (%)	week 2 (*N* = 43) n (%)	*p*-value^a^
ECOG status			1.00
0	81 (57.0)	25 (58.1)	
1	60 (42.3)	18 (41.9)	
Missing	1	0	
KRAS status			0.023
Wildtype	81 (57.0)	16 (37.2)	
Mutant	61 (43.0)	27 (62.8)	
Alkaline Phosphatase ≥ site ULN	69 (49.3)	15 (35.7)	0.158
Total bilirubin ≥ site ULN	11 (7.8)	2 (4.8)	0.736
CEA ≥ 35 ng/mL	80 (58.8)	20 (46.5)	0.164
Albumin ≥ site LLN	124 (89.9)	35 (83.3)	0.275
Bilobar disease	112 (78.9)	39 (90.7)	0.079
Liver tumour burden			0.660
< 10%	86 (61.0)	28 (65.1)	
≥ 10% to 25%	37 (26.2)	12 (27.9)	
≥ 25%	18 (12.8)	3 (7.0)	
Missing	1		
Maximum liver lesion size ≥ 40 mm	115 (81.0)	35 (81.4)	0.952
Primary tumour in situ	53 (37.3)	14 (32.6)	0.569
Left side primary tumour location	100 (70.9)	30 (73.2)	0.846
Extrahepatic lesions at baseline	73 (51.4)	23 (53.5)	0.811
Number of lesions			0.422
< 3	13 (9.2)	8 (18.6)	
3–5	30 (21.3)	8 (18.6)	
6–10	40 (28.4)	10 (23.3)	
> 10	58 (41.1)	17 (39.5)	
Missing	1	0	

^a^ By Fisher’s exact test

CEA, carcinoembryonic antigen; LLN, lower limit of normal; ULN, upper limit of normal

Treatment was inclusive of first week Wednesday to second week Tuesday for most patients in EPOCH (133/187; 71.9%). Patients who received more than one vial included post calibration days of week 1 only (*n* = 103, 55.7%), week 2 only (*n* = 38, 20.5%) or a combination of week 1 and week 2 (*n* = 44, 23.8%). EPOCH patients were assigned to week 1 (*n* = 142, 76.8%) or week 2 (*n* = 43, 23.2%) based on the vial used to infuse the largest perfused volume.

Median and mean ± SD perfused volume absorbed dose was 117 Gy and 115.5 ± 10.7 Gy, respectively. Perfused volume absorbed dose for week 1 was a median of 116.6 Gy (range: 61.7, 156.0) with a mean of 115.5 ± 10.4 Gy and week 2 was a median of 118.1 Gy (range: 68.2, 133.7) with a mean ± SD of 115.5 ± 11.5 Gy, and were comparable by 2-sample t-test (*p* = 0.98).

No statistical difference in ORR, PFS, hPFS or OS were noted for week 1 versus week 2 (Table 7 and Supplementary Fig. 3).

**Table 7:** EPOCH Tumour response, PFS, hPFS and OS for week 1 and week 2

	week 1 (*N* = 142)	week 2 (*N* = 43)
Objective response rate* (%)	38.0 (95% CI: 30.5, 46.2)]	34.9 (95% CI: 22.4, 49.8)
	Difference: 3.1 percentage points (95% CI: −14.8, 19.1), *p* = 0.71	
Median PFS* (months)	9.0 (95% Cl: 7.2, 9.4)	7.8 (95% Cl: 5.6, 10.9)
	HR: 0.99 (95% Cl: 0.64, 1.52), *p* = 0.95	
Median hPFS* (months)	9.2 (95% CI: 7.9, 9.8)	8.8 (95% CI: 7.0, 12.9)
	HR: 1.07 (95% CI: 0.68, 1.71), *p* = 0.76	
Median OS (months)	14.9 (95% CI: 11.7, 15.8)	16.4 (95% CI: 11.6, 19.5)
	HR: 1.19 (95% CI: 0.81, 1.74), *p* = 0.37	

*using RECIST 1.1 by blinded independent central review; p-values by Cox regression for PFS, hPFS and OS, and by continuity adjusted Newcombe-Wilson approach for objective response rate

hPFS, hepatic progression-free survival; HR, hazard ratio; OS, overall survival; PFS, progression-free survival

Median OS for week 1 (54/142) and week 2 (15/43) responders was 23.6 months (95% CI: 17.5, 27.1) and 19.1 months (95% CI: 12.9, 44.8), respectively (Supplementary Fig. 4). OS was not significantly different when comparing week 1 to week 2 responders by Cox regression, *p* = 0.43; HR = 1.33 (95% CI: 0.66, 2.70).

Although baseline CEA levels were higher for week 1, the percent reduction was comparable for week 1 and week 2 in patients with an abnormal CEA level (> 5 ng/mL) following treatment with ^90^Y glass microspheres plus systemic chemotherapy (Table 8).

**Table 8:** EPOCH Baseline CEA levels and percent change in CEA levels for patients with an abnormal baseline CEA (> 5 ng/mL) for week 1 and 2

	week 1 (*N* = 120)		week 2 (*N* = 37)		
	Median	Mean (SD)	Median	Mean (SD)	*p*-value^a^
Baseline CEA (ng/mL)	89.1	711.8 (2298.9)	40.8	507.5 (1305.5)	0.22
Median CEA change (%)	−77.8	−57.3 (54.6)	−79.6	−46.4 (83.9)	0.51

^a^ By Mann–Whitney U test

CEA, carcinoembryonic antigen; SD, standard deviation

In patients with an abnormal baseline CEA (> 5 ng/mL), ORR was 38.3% and 37.8% for weeks 1 and 2, respectively, *p* = 0.89 (by continuity adjusted Newcombe-Wilson approach). While week 1 patients had a higher percentage of patients with a median baseline CEA level ≥ 35 ng/mL, there was not a statistical difference in the number of week 1 patients achieving either a ≥ 25% (*p* = 0.90) or ≥ 50% (*p* = 0.93) decrease in baseline CEA levels versus week 2 patients (Supplementary Table 4). In patients experiencing a ≥ 50% reduction in baseline CEA levels, median OS was 17.5 months for week 1 and 17.7 for week 2, HR = 1.18 (95% CI: 0.70, 1.99), *p* = 0.54 by Cox regression.

Patients receiving week 1 treatment had fewer ≥ grade 3 device related asthenia (9.2% versus 23.3%; *p* = 0.014), which is commonly reported for radioembolization, and gastrointestinal hemorrhage (0% versus 7.0%; *p* = 0.012), with no known association with radioembolization and/or microsphere number, than week 2 (Table 9). All device-related AEs of interest for week 1 and 2 are reported in Supplementary Table 5.

**Table 9:** EPOCH device-related Adverse Events of interest for week 1 and 2

AEs (n, %)	week 1 (*N* = 142)	week 2 (*N* = 43)	*p*-value^a^
All Grade 3 or 4 AEs^b^	96 (67.6)	31 (72.1)	0.578
Granulocytopenia	36 (25.4)	13 (30.2)	0.525
Abdominal discomfort	12 (8.5)	2 (4.7)	0.527
Anemia	9 (6.3)	4 (9.3)	0.504
Asthenia	13 (9.2)	10 (23.3)	0.014
Gastrointestinal hemorrhage	0	3 (7.0)	0.012
Lymphocyte count decreased	5 (3.5)	3 (7.0)	0.391

^a^ By Fisher’s exact test

^b^ All ≥ grade 3 AEs occurring in ≥ 5% of patients (either week 1 or week 2)

## Discussion

Three variables are inherent in the selection of treatment day for ^90^Y glass microsphere administration: desired absorbed dose (Gy), specific activity per microsphere, and number of microspheres, and by selecting two variables, the third is set. The number of microspheres can vary by approximately 17-fold over the 12-day shelf life. As illustrated in Figs. 1 and 2, the simulated distributions at an identical mean TAD are heterogeneous. In a single center study, the mean HI for TAD was 7 (standard deviation of 6.7; range: 0.01 to 35) in patients treated with either ^90^Y glass or resin microspheres [5]. In contrast, the HI for stereotactic radiosurgery is < 2, while other forms of external beam radiation approach 1. Computational data demonstrates more homogenous particle coverage could result in radioembolization therapeutic benefit, but there is a lack of published clinical data supporting this hypothesis [4, 8, 11]. The computational modelling illustrated in Figs. 1 and 2 and Table 2 were based on clinically derived HI and illustrate the heterogenous distribution within tumour and normal tissue noted in post ^90^Y-radioembolization DVH. DVH curves note increasing activity as the key driver to achieve higher TAD (mean, D95, D5) and reduce or eliminate tumour volume that does not achieve tumoricidal absorbed dose, i.e., volumes achieving absorbed dose > 100 Gy (V_0–100_). Similarly, the model illustrates that TAD V_0–100_ and NTAD V30 exhibit limited changes based on week 1 or week 2 treatment and increasing microsphere number for an equivalent activity and T:N ratio (Table 2, Examples A-D). However, increasing the activity administered yields a notable reduction TAD V_0–100_ and an associated increase in NTAD V30. TARGET and EPOCH allowed physician selection for treatment day with comparable target absorbed dose allowing the impact of microsphere number to be evaluated relative to efficacy and safety outcomes. Notably in TARGET and EPOCH, an increase in the number of particles occurring on later treatment days, relative to the day of calibration, did not demonstrate an increase in efficacy outcomes. The ≥ grade 3 AE profiles were comparable in TARGET, with an increased frequency and severity of select AEs in EPOCH, i.e., asthenia.

TARGET and EPOCH are the first large patient datasets sufficient to allow subgroup comparison of treatment week impact on outcomes in patients undergoing treatment with ^90^Y glass microspheres. Analysis of the TARGET and EPOCH data demonstrated no differences for dosimetry for weeks 1 and 2, and no statistical difference in efficacy treatment outcomes by treatment week (ORR, tumour marker response [AFP for TARGET; CEA for EPOCH], PFS/hPFS [EPOCH only] and OS). The theoretical efficacy benefit by increasing the number of ^90^Y microspheres to theoretically obtain a more homogenous distribution was not demonstrated. Possible reasons for not observing a benefit include a higher impact of TAD or D_70_ versus a theoretical more homogenous distribution, differences in specific activity within the allocated week, heterogenous microsphere distribution in the tumour, influence of tumour size and tumour biology [5, 8]. These results are also in line with previous studies showing that above a threshold of ~ 10,000 microspheres, DVH curves showed minimal changes with additional microspheres, confirming that TAD and specific activity are more important variables for improved patient outcomes [6]. Indeed, in recent studies, median specific activity and tumour dose but not number of particles were significantly associated with CPN [18, 19], and higher V100 (i.e., tumour dose) was associated with improved treatment response (e.g., CR, PFS, OS) [5, 7].

The more heterogenous distribution of ^90^Y glass microspheres is credited with preservation of normal liver tissue, with a higher tolerated NTAD as the number of microspheres is decreased [10]. In a normal porcine liver study evaluating normal tissue distribution, the fraction of approximated lobules with an NTAD ≥ 30 Gy at the same mean perfused volume absorbed dose was 28.7%, 41.7% and 64.3% at treatment days 4, 8 and 12 post-calibration, respectively. At later treatment days, the number of ^90^Y glass microspheres clusters/mL increased, distance between clusters decreased, while cluster size was similar for day 4, day 8 and day 12 and approximately doubled for day 16. These findings suggest a higher overall normal liver parenchyma exposure, more homogenous particle coverage, as the number of microspheres increases.

A more homogenous distribution of microspheres and a higher NTAD in normal liver tissue may also occur as a result of a flow-directed decrease in the T:N ratio when higher numbers of ^90^Y microspheres are delivered [6, 8, 13, 20]. A decrease in the T:N ratio disproportionally results in a higher NTAD because of “overflow” into normal liver parenchyma. When overflow occurs, the predictability of pretreatment [^99 m^Tc]Tc-MAA dosimetry estimation is reduced for TAD and NTAD [21]. No evidence of overflow was noted in TARGET or EPOCH, which may be a result of the (relative early) treatment day relative to calibration for the majority of patients, the hypervascularity of HCC tumours and the relatively low protocol specified perfused volume absorbed dose of 120 Gy ± 10% for EPOCH.

Dosimetry for ^90^Y glass microspheres treatment has evolved with numerous retrospective and prospective clinical studies demonstrating the impact of TAD on patient efficacy outcomes [7, 12, 15, 22]. A recent update to HCC recommendations notes a preference, based on current clinical data, to administer ^90^Y glass microspheres between week 1 Wednesday up to and including week 2 Tuesday [12]. The recommended treatment days are based on both single and multi-institution prospective and retrospective datasets that used ^90^Y glass microspheres within this range. While emerging data suggests early treatment, specifically for high absorbed dose radiation segmentectomy which may increase CPN, an optimal treatment day (i.e., specific activity) has not been established [5, 12, 15–17, 19, 23]. An example of effective week 1 treatment of large tumours using both a high TAD and specific activity, is the DOSISPHERE randomized controlled trial [16, 24]. DOSISPHERE treated large HCC tumours (mean target lesion ≥ 10.5 cm) and demonstrated a tumour response of 71% by EASL criteria and median OS of 26.6 months in predominantly BCLC-C HCC patients with an acceptable safety profile.

Correlation of the [^99 m^Tc]Tc-MAA and ^90^Y glass microspheres distribution is closely dependent on reproducing catheter tip position, and an increasing number of publications have demonstrated the utility of [^99 m^Tc]Tc-MAA as a surrogate with appropriate catheter technique [25, 26]. One aspect of the technique crucial to predictability is the number of ^90^Y glass microspheres. A comparison of week 1 day four with week 2 day eight ^90^Y glass microsphere treatment noted a trend towards better predictability of [^99 m^Tc]Tc-MAA as a surrogate for week 1 day four [21].

The extent of heterogenous distribution and the target TAD for ^90^Y glass and resin microspheres are different [27–29]. This heterogeneity is reflected in DVH analysis of tumours, where D_70_ is often cited as a predictor of tumour response. In biopsy samples in mCRC patients treated with either ^90^Y glass or resin microspheres, heterogenous distribution of both types was noted within the tumour [29]. Analysis noted distances varying from 0–6 mm between microspheres where 5/86 specimens noted a single ^90^Y microsphere. Equivalent uniform dose (EUD) attempts to overcome these differences by correcting for dose distribution, but with inherent limitations. In a comparative study, a 118 Gy mean EUD-based TAD was noted as an effective dose minimum for ^90^Y glass and resin microsphere [4, 9]. The large variability in HI for radioembolization, however, does not allow for accurate correlation between EUD and patient outcomes, rather optimal dosimetry should focus on dose thresholds for each product separately [5, 15, 17, 30–34]. A better prediction of tumour response may be accomplished through mean TAD and DVH analysis [4, 5]. DVH often cite a specific value, i.e., D_70_, where DVH analysis highlights the heterogeneity of deposition and improved outcomes when a minimum absorbed dose to a specified tumour volume is achieved, e.g., the volume of tumour receiving ≥ 100 Gy, V_0–100_ [4, 5, 7]. Heterogeneity of deposition was cited as a potential contributor for individual patient differences in DVH analysis versus the mean TAD for both glass and resin ^90^Y microspheres, which is reflected in the HI [3–5]. Evaluation of HI utilizing post ^90^Y SPECT/CT was not a statistically significant predictor of ORR or CR by either univariate or multivariate analysis [5]. If HI was associated with efficacy outcomes it may support theoretical homogenous distribution as critical to efficacy outcomes, however, this conclusion was not reached. In line with published clinical data, D_70_ was associated with efficacy outcomes by multivariate analysis [5]. Moreover, the volume of underdosed tumour based on post ^90^Y dosimetry, defined as the volume receiving < 100 Gy, was the only significant dosimetric predictor for CR and OS [5]. In a single institution study in large tumours (11.4 ± 3.9 cm) with TARE, ablative dosimetry defined as > 150 Gy to the liver (242.3 ± 63.6 Gy to the perfused volume), high TAD (455.2 ± 261.3 Gy) demonstrated CPN in 4/7 surgical specimens. Treatment was administered as week 1 (60%) or week 2 (40%) following the current recommendations. A higher CPN rate was noted for patients with a higher mean TAD, 712.9 ± 316.6 Gy versus 337.1 ± 149.8 Gy for noncomplete necrosis. By multivariate logistic regression analysis only V_0–100_ was significantly associated with CR [7]. Predictability of mean TAD or specific DVH values requires additional clinical data as published literature currently reflects conflicting results in small patient datasets regarding improved predictability of DVH versus mean TAD [4, 5, 7, 21, 33].

A factor noted in published literature, but for which this publication’s authors disagree, is that there is an upper limit to the TAD to achieve optimal patient outcomes [7, 15, 30, 32–34]. For ^90^Y glass microspheres, an upper limit TAD has not been identified in personalized dosimetry, i.e., radiation segmentectomy using single compartment dosimetry or in lobar treatment with multicompartment dosimetry [12, 35]. A strong correlation exists between TAD and improved patient outcomes for ^90^Y glass and resin microspheres [35, 36]. As TAD increases, the D_70_ will shift to the right in a DVH curve, which will also lead to a reduction in the percent tumour with a V_0–100_.

Limitations of the presented data include a lack of clinical data to inform microsphere distribution modelling, post-hoc analysis, non-randomization of week 1 and week 2 treatment, EPOCH patients receiving week 1 only, week 2 only and combinations of week 1 and week 2 vials, dissimilar endpoints and level of safety assessment for TARGET, lack of multicompartment dosimetry for EPOCH, lack of standardized methodology in selecting week 1 versus 2 in EPOCH, no direct comparison with other products and heterogeneity of studied populations and tumour types, as well as the focus on mRECIST for HCC and RECIST for mCRC.

To further optimize patient outcomes, future studies and publications should include dosimetry details, i.e., treatment day, specific activity, administered microsphere number, TAD (mean and DVH) and NTAD for each product utilized. Collected data will allow associations with each dosimetry factor to be independently assessed and their relative contributions to patient outcomes assigned.

## Conclusions

Analysis of TARGET and EPOCH study populations did not identify an efficacy treatment benefit for week 2 ^90^Y glass microsphere treatment versus week 1 treatment but did suggest that infusion at later treatment days (i.e., week 2) resulted in a higher rate and severity of minor AEs. Instead, TAD was confirmed as the primary driver for patient efficacy outcomes.

## Supplementary Information

Below is the link to the electronic supplementary material.

Supplementary file1 (PDF 483 KB)

## Data Availability

Data will be available upon reasonable request.
