# Supplementary material for: The relationship between yttrium-90 glass microspheres specific activity, particle density and treatment outcomes in HCC and mCRC
Source: Eur J Nucl Med Mol Imaging. 2025 May 22;52(13):4834–46. doi: 10.1007/s00259-025-07334-8 (PMC12589197; doi:10.1007/s00259-025-07334-8)
Supplement: Supplementary file 1 — Supplementary file1 (PDF 483 KB) [file 259_2025_7334_MOESM1_ESM.pdf]

Title: The relationship between yttrium-90 glass microspheres specific activity, particle density and treatment outcomes in HCC and mCRC

Authors: Marnix GEH Lam[1*], Etienne Garin[2], Kirk Fowers[3], Armeen Mahvash[4], Siddharth A Padia[5], and Riad Salem[6]

Supplementary Figures and Tables

Supplementary Figure 1: TARGET Kaplan Meier OS for week 1 and week 2.

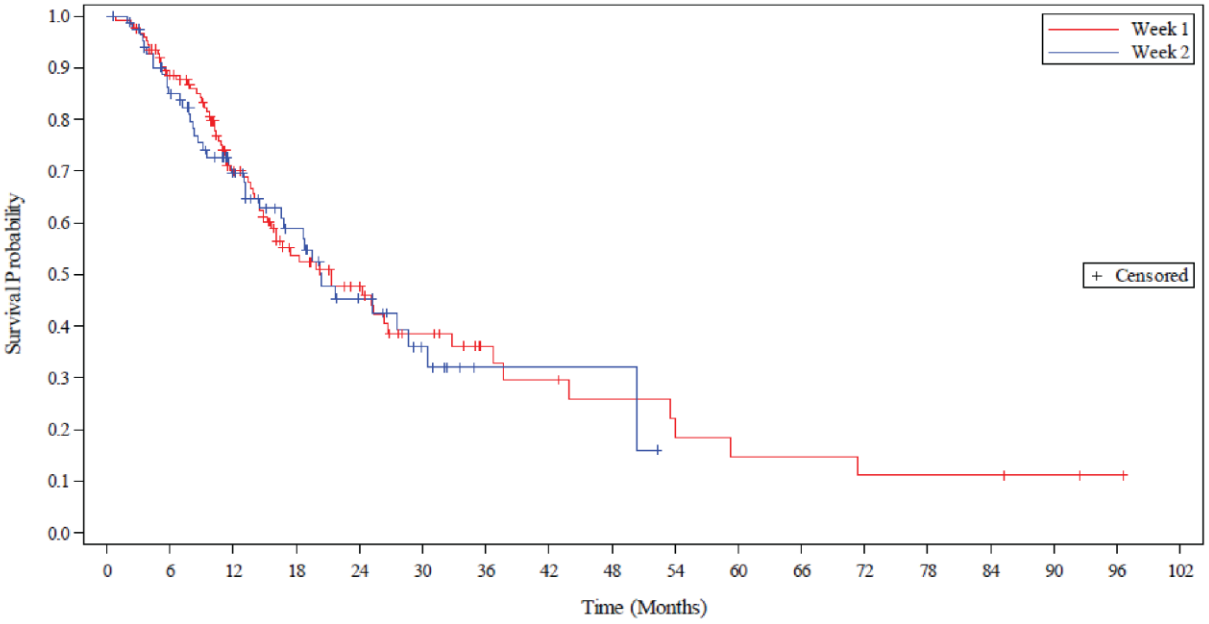

N at Risk

Week 1	125	104	69	38	28	18	11	9	7	5	4	4	3	3	3	2	1	0
Week 2	84	66	44	29	17	9	2	2	2	0	0	0	0	0	0	0	0	0

Supplementary Figure 2: TARGET Kaplan Meier OS for week 1 (Mon-Wed), week 1 (Thu-Sun), week 2 (Mon-Tue) and week 2 (Wed-Fri)

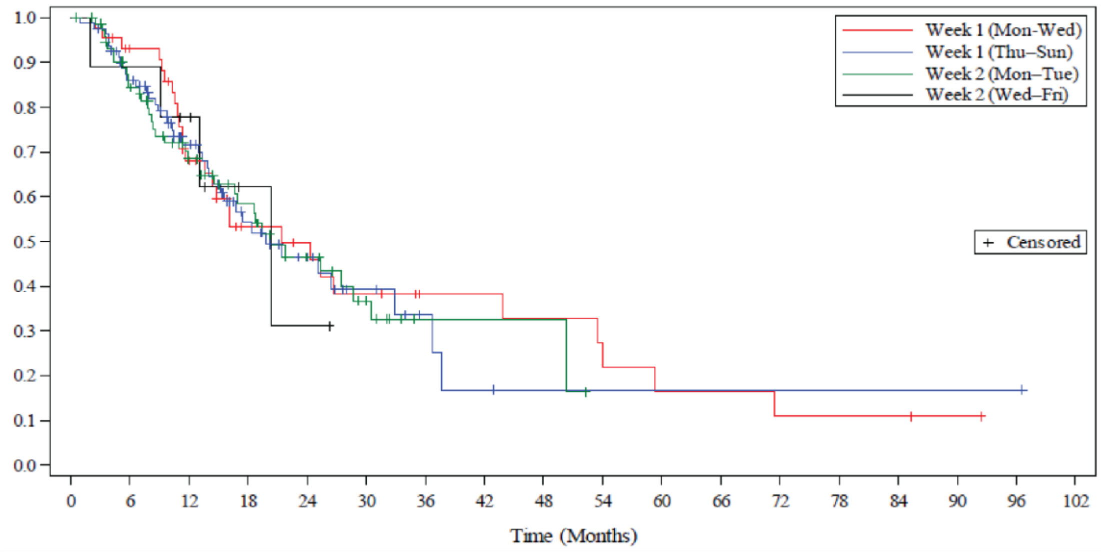

N at Risk

Week 1 (Mon–Wed)	44	38	26	15	13	10	7	7	6	4	3	3	2	2	2	1	0	0
Week 1 (Thu–Sun)	81	66	43	23	15	8	4	2	1	1	1	1	1	1	1	1	1	0
Week 2 (Mon–Tue)	75	58	38	27	16	9	2	2	2	0	0	0	0	0	0	0	0	0
Week 2 (Wed–Fri)	9	8	6	2	1	0	0	0	0	0	0	0	0	0	0	0	0	0

Supplementary Figure 3: EPOCH Kaplan-Meier curves of OS comparing ^{90}Y glass microspheres week 1 versus week 2 plus systemic chemotherapy.

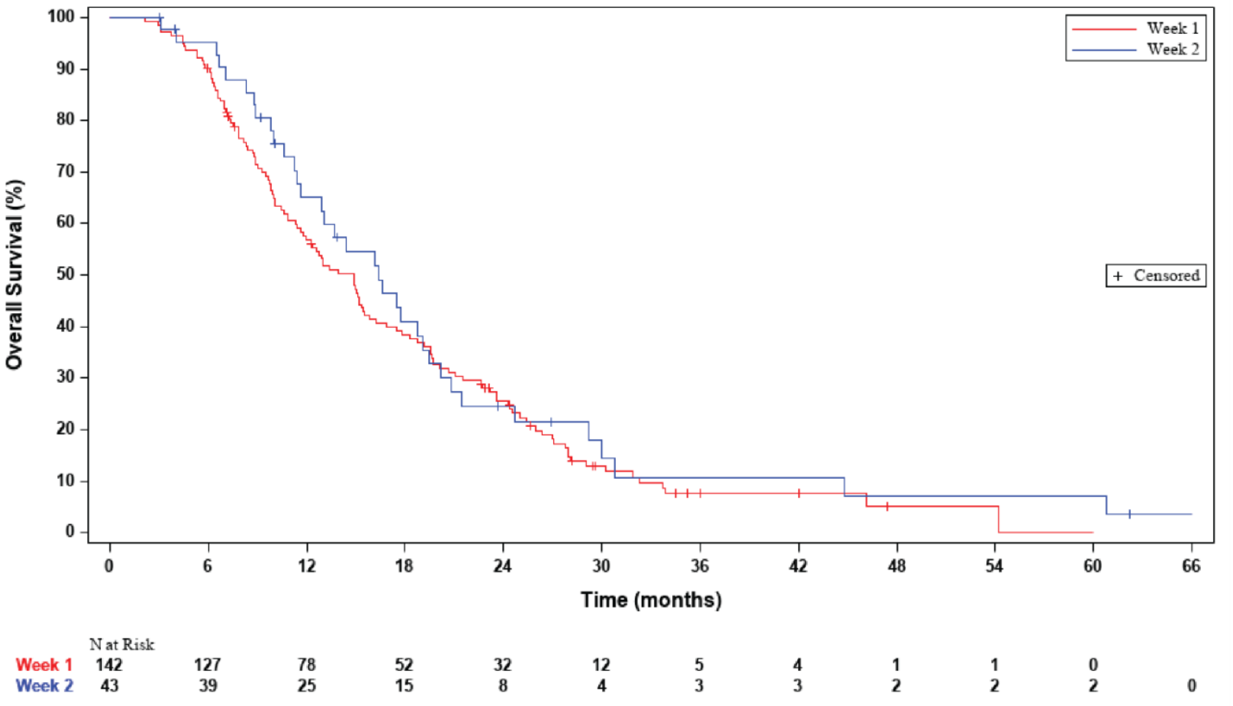

Supplementary Figure 4: EPOCH Kaplan-Meier OS curves comparing ^{90}Y glass microspheres week 1 vs week 2 plus systemic chemotherapy in RECIST 1.1 responders by blinded independent central review.

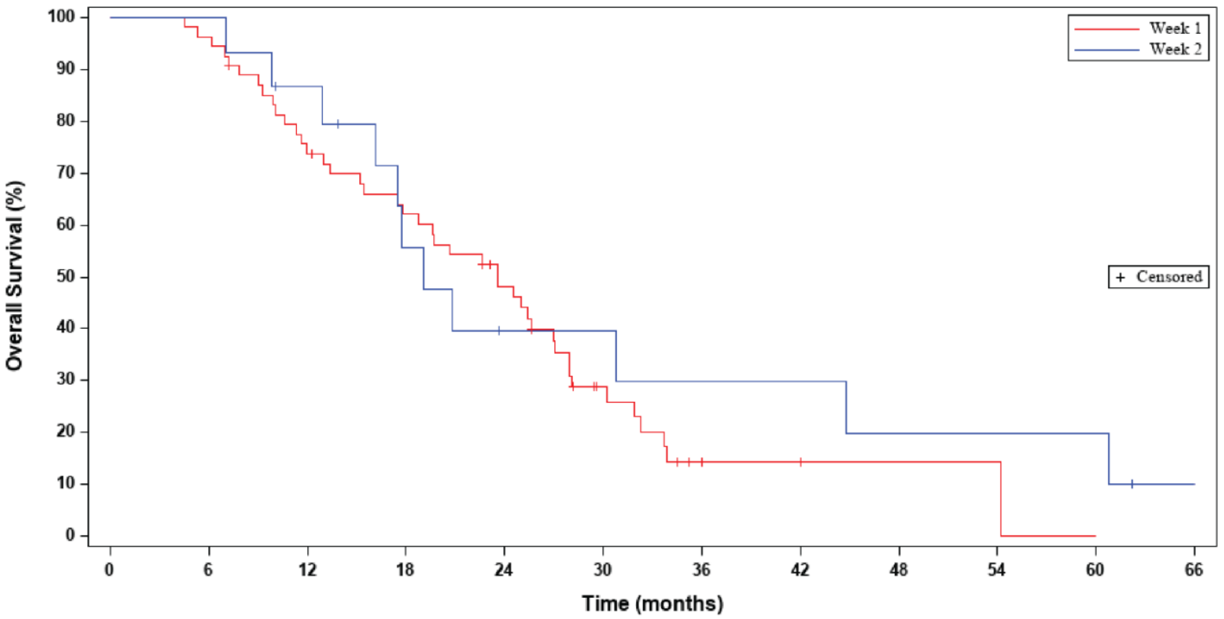

N at Risk											
Week 1	54	52	39	32	23	10	3	2	1	1	0	
Week 2	15	15	12	7	4	4	3	3	2	2	2	0

Supplementary Table 1: TARGET patient and disease characteristics (week 1 Mon – Wed/Thu – Sun and week 2 Mon – Tue/Wed – Fri)

	week 1 Mon – Wed (N=44), n (%)	week 1 Thu – Sun (N=81), n (%)	week 2 Mon – Tue (N=75), n (%)	week 2 Wed – Fri (N=9), n (%)	p-value[a]
ECOG status					0.506[b]
0	31 (70.5)	51 (63.0)	49 (65.3)	4 (44.4)	
1	12 (27.3)	28 (34.6)	24 (32.0)	3 (33.3)	
2/3	1 (2.3)	2 (2.5)	2 (2.7)	2 (22.2)	
BCLC stage					0.932
A	5 (11.4)	13 (16.0)	8 (10.7)	1 (11.1)	
B	15 (34.1)	24 (29.6)	27 (36.0)	2 (22.2)	
C	24 (54.5)	44 (54.3)	40 (53.3)	6 (66.7)	
Child-Pugh status					0.318
A (5-6)	39 (88.6)	76 (93.8)	64 (85.3)	8 (88.9)	
B7	4 (11.4)	5 (6.2)	11 (14.7)	1 (11.1)	
Unilobar	27 (61.4)	58 (71.6)	57 (76.0)	6 (66.7)	0.384
Bilobar	17 (38.6)	23 (28.4)	18 (24.0)	3 (33.3)	
Presence of PVT	17 (38.6)	22 (27.2)	28 (37.3)	2 (22.2)	0.402
Bilirubin					0.785
<1.0 mg/dL	30 (68.2)	52 (64.2)	53 (70.7)	7 (77.8)	
≥1.0 mg/dL	14 (31.8)	29 (35.8)	22 (29.3)	2 (22.2)	
Albumin					0.367
<3.6 g/dL	14 (31.8)	27 (33.3)	34 (45.3)	3 (33.3)	
≥3.6 g/dL	30 (68.2)	54 (66.7)	41 (54.7)	6 (66.7)	
Ascites					0.487
Absent	41 (93.2)	74 (91.4)	64 (85.3)	8 (88.9)	
Slight	3 (6.8)	7 (8.6)	11 (14.7)	1 (11.1)	
HCC Etiology					
Alcohol	16 (36.4)	19 (23.5)	13 (17.3)	0	0.040
Hepatitis C	10 (22.7)	21 (25.9)	36 (48.0)	2 (22.2)	0.008
Unknown etiology	9 (20.5)	14 (17.3)	8 (10.7)	1 (11.1)	0.462
Hepatitis B	5 (11.4)	9 (11.1)	15 (20.0)	2 (22.2)	0.321
Non-alcoholic steatohepatitis	3 (6.8)	10 (12.3)	5 (6.7)	2 (22.2)	0.263
Other	1 (2.3)	7 (8.6)	4 (5.3)	1 (11.1)	0.362

[a] By Fisher's exact test

[b] Statistical analysis compared weeks 1 and 2 for ECOG 0 and ECOG ≥1

Abbreviations: BCLC, Barcelona Clinic Liver Cancer; ECOG, Eastern Cooperative Oncology Group; HCC, hepatocellular carcinoma; n, number of patients per category; N, total number of patients per group; PVT, portal vein thrombosis; TARGET, The **T**heraSphere **A**dvanced Dosimetry **R**etrospective **G**lobal Study **E**valuation in Hepatocellular Carcinoma **T**reatment

Supplementary Table 2: TARGET median OS for week 1 (Mon-Wed), week 1 (Thu-Sun), week 2 (Mon-Tue) and week 2 (Wed-Fri)

	Median OS months (95% CI)	p-value[a]
week 1 (Mon-Wed), N=44	21.4 (13.6, 53.6)	0.732
week 1 (Thu-Sun), N=81	19.8 (14.6, 32.9)	
week 2 (Mon-Tue), N=75	20.2 (14.5, 30.5)	
week 2 (Wed -Fri), N=9	20.3 (2.0, NE)	

[a] by both log rank test and Cox regression

Abbreviations: CI, confidence interval; N, total number of patients per group; NE, not estimable; OS, overall survival; TARGET, The **T**heraSphere **A**dvanced Dosimetry **R**etrospective **G**lobal Study **E**valuation in Hepatocellular Carcinoma **T**reatment

Supplementary Table 3: TARGET device related ≥Grade 3 AEs by days of treatment

AEs[a] (n, %)	week 1 Mon-Wed (N=44)	week 1 Thu-Sun (N=81)	week 2 Mon-Tue (N=75)	week 2 Wed-Fri (N=9)	p-value[b]
Patients with device related AEs of ≥grade 3	6 (13.6)	15 (18.5)	20 (26.7)	2 (22.2)	0.359
Abdominal pain	0	0	2 (2.7)	0	0.256
Ascites	0	3 (3.7)	7 (9.3)	0	0.134
Blood bilirubin increased	4 (9.1)	4 (4.9)	1 (1.3)	0	0.222
Lymphopenia	3 (6.8)	6 (7.4)	9 (12.0)	1 (11.1)	0.644
Oedema	0	1 (1.2)	2 (2.7)	0	0.660
Thrombocytopenia	0	1 (1.2)	3 (4.0)	0	0.460

[a] Adverse events in 2 or more patients (either week 1 or week 2) were included

[b] By Fisher's exact test

Abbreviations: AE, adverse event; n, number of patients per category; N, total number of patients per group; TARGET, The **T**heraSphere **A**dvanced Dosimetry **R**etrospective **G**lobal Study **E**valuation in Hepatocellular Carcinoma **T**reatment

Supplementary Table 4: EPOCH Baseline CEA levels and percent change in CEA levels by percent decrease in baseline CEA for patients with an abnormal baseline CEA (>5 ng/mL)

	week 1 (N=142)		week 2 (N=43)	
	Baseline CEA decrease by ≥25 % (n=95)	Baseline CEA decrease by ≥50 % (n=79)	Baseline CEA decrease by ≥25 % (n=29)	Baseline CEA decrease by ≥50 % (n=24)
Baseline CEA <35 ng/mL, n (%)	32 (33.7)*	25 (31.6)**	15 (51.7)*	11 (48.5)**
Baseline CEA ≥35 ng/mL, n (%)	63 (66.3)*	54 (68.4)**	14 (48.3)*	13 (54.2)**
ORR, n (%)	43 (45.3)	37 (46.8)	14 (48.3)	12 (50.0)

*p=0. 90 comparing week 1 vs week 2 by continuity adjusted Wald approach
**p=0.93 comparing week 1 vs week 2 by continuity adjusted Wald approach

Abbreviations: CEA, carcinoembryonic antigen; EPOCH, **E**valuating TheraSphere in **P**atients with metastatic colorectal carcinoma **O**f the Liver who have progressed on first-line **Ch**emotherapy; n, number of patients per category; N, total number of patients per group; ORR, objective response rate

Supplementary Table 5: EPOCH device related Adverse Events of interest for week 1 and 2

AEs (n, %)	week 1 (N=142)	week 2 (N=43)	p-value[a]
All AEs[b]	138 (97.2)	42 (97.7)	1.000
Abdominal discomfort	61 (43.0)	27 (62.8)	0.023
Alopecia	15 (10.6)	8 (18.6)	0.162
Anemia	18 (12.7)	9 (20.9)	0.179
Ascites	13 (9.2)	5 (11.6)	0.572
Aspartate Aminotransferase increased	21 (14.8)	4 (9.3)	0.357
Asthenia	85 (59.9%)	28 (65.1%)	0.536
Blood bilirubin increased	11 (7.7)	8 (18.6)	0.049
Blood pressure fluctuation	16 (11.3)	4 (9.3)	1.000
Catheter site erythema	14 (9.9)	6 (14.0)	0.416
Constipation	41 (28.9)	19 (44.2)	0.060
Cough	9 (6.3)	6 (14.0)	0.119
Decreased appetite	36 (25.4)	8 (18.6)	0.363
Dermatitis	7 (4.9)	3 (18.6)	0.154
Diarrhea	46 (32.4)	13 (30.2)	0.790
Dizziness	11 (7.7)	7 (16.3)	0.138
Dyspepsia	19 (13.4)	4 (9.3)	0.478
Gastrointestinal disorder	4 (2.8)	9 (20.9)	<0.001
Granulocytopenia	58 (40.8)	16 (37.2)	0.670
Headache	7 (4.9)	8 (18.6)	0.008
Insomnia	10 (7.0)	6 (14.0)	0.212
Leukopenia	11 (7.7)	5 (11.6)	0.535
Lymphocyte count decreased	10 (7.0)	6 (14.0)	0.212
Mouth ulceration	19 (13.4)	3 (7.0)	0.256
Nausea	60 (42.3)	24 (55.8)	0.118
Neuropathy peripheral	42 (29.6)	6 (14.0)	0.041
Platelet count decreased	38 (26.8)	10 (23.3)	0.646
Pyrexia	28 (19.7)	11 (25.6)	0.409
Rash	18 (12.7)	7 (16.3)	0.545
Stomatitis	16 (11.3)	4 (9.3)	1.000
Urinary tract infection	8 (5.6)	5 (11.6)	0.184
Vomiting	33 (23.2)	9 (20.9)	0.752

[a] By Fisher's exact test

[b] All grade AEs occurring in ≥10% of patients (either week 1 or week 2)

Abbreviations: AE, adverse event; EPOCH, **E**valuating TheraSphere in **P**atients with metastatic colorectal carcinoma **O**f the Liver who have progressed on first-line **Ch**emotherapy; n, number of patients per category; N, total number of patients per group
